# Iceland screens, treats, or prevents multiple myeloma (iStopMM): a population-based screening study for monoclonal gammopathy of undetermined significance and randomized controlled trial of follow-up strategies

**DOI:** 10.1038/s41408-021-00480-w

**Published:** 2021-05-17

**Authors:** Sæmundur Rögnvaldsson, Thorvardur Jon Love, Sigrun Thorsteinsdottir, Elín Ruth Reed, Jón Þórir Óskarsson, Íris Pétursdóttir, Guðrún Ásta Sigurðardóttir, Brynjar Viðarsson, Páll Torfi Önundarson, Bjarni A. Agnarsson, Margrét Sigurðardóttir, Ingunn Þorsteinsdóttir, Ísleifur Ólafsson, Ásdís Rósa Þórðardóttir, Elías Eyþórsson, Ásbjörn Jónsson, Andri S. Björnsson, Gunnar Þór Gunnarsson, Runólfur Pálsson, Ólafur Skúli Indriðason, Gauti Kjartan Gíslason, Andri Ólafsson, Guðlaug Katrín Hákonardóttir, Manje Brinkhuis, Sara Lovísa Halldórsdóttir, Tinna Laufey Ásgeirsdóttir, Hlíf Steingrímsdóttir, Ragnar Danielsen, Inga Dröfn Wessman, Petros Kampanis, Malin Hultcrantz, Brian G. M. Durie, Stephen Harding, Ola Landgren, Sigurður Yngvi Kristinsson

**Affiliations:** 1 Faculty of Medicine, Univeristy of Iceland, Reykjavík, Iceland; 2 Dept of Hematology, Rigshospitalet, Copenhagen Denmark; 3 Landspítali University Hospital, Reykjavík, Iceland; 4 Faculty of Psychology, University of Iceland, Reykjavik, Iceland; 5 Akureyri Hospital, Akureyri, Iceland; 6 Faculty of Economics, University of Iceland, Reykjavik, Iceland; 7 The Binding Site, Birmingham, West Midlands UK; 8 Memorial Sloan Kettering Cancer Center, New York, NY USA; 9 Cedar-Sinai Samual Oschin Cancer Center, Los Angeles, CA USA; 10 Sylvester Comprehensive Cancer Center, University of Miami, Miami, FL USA

**Keywords:** Randomized controlled trials, Myeloma

## Abstract

Monoclonal gammopathy of undetermined significance (MGUS) precedes multiple myeloma (MM). Population-based screening for MGUS could identify candidates for early treatment in MM. Here we describe the Iceland Screens, Treats, or Prevents Multiple Myeloma study (iStopMM), the first population-based screening study for MGUS including a randomized trial of follow-up strategies. Icelandic residents born before 1976 were offered participation. Blood samples are collected alongside blood sampling in the Icelandic healthcare system. Participants with MGUS are randomized to three study arms. Arm 1 is not contacted, arm 2 follows current guidelines, and arm 3 follows a more intensive strategy. Participants who progress are offered early treatment. Samples are collected longitudinally from arms 2 and 3 for the study biobank. All participants repeatedly answer questionnaires on various exposures and outcomes including quality of life and psychiatric health. National registries on health are cross-linked to all participants. Of the 148,704 individuals in the target population, 80 759 (54.3%) provided informed consent for participation. With a very high participation rate, the data from the iStopMM study will answer important questions on MGUS, including potentials harms and benefits of screening. The study can lead to a paradigm shift in MM therapy towards screening and early therapy.

## Introduction

Monoclonal gammopathy of undetermined significance (MGUS) is characterized by the presence of monoclonal immunoglobulins (M proteins) or an abnormal ratio of free immunoglobulin light chains (FLC) in the blood^1^. MGUS can be classified by the type of M proteins present. Non-IgM MGUS is the most common type and is defined by the presence of IgG, IgA, and rarely IgD or IgE M proteins^2^. IgM MGUS is defined by the presence of IgM M proteins^3^. Light-chain (LC) MGUS is defined by an abnormal FLC-ratio, indicating an excess of monoclonal FLCs in the absence of M proteins^4^. Non-IgM MGUS and LC-MGUS are caused by monoclonal bone marrow plasma cells (BMPCs) and are the precursor of multiple myeloma (MM), a malignancy of BMPCs^5,6^. IgM MGUS is caused by monoclonal lymphoplasmacytic lymphocytes and is a precursor to other lymphoproliferative disorders (LP), most notably Waldenström’s macroglobulinemia (WM), and rarely MM^3^. In addition, MGUS of all types, especially LC-MGUS, can precede amyloid light chain amyloidosis (AL)^7^. Prior studies suggest a 1% annual risk of progressing from MGUS and LC-MGUS to frank malignancy^1,3,4,8^.

Before progressing to MM or WM, MGUS is believed to pass through a smoldering MM or WM phase (SMM and SWM), which is associated with a higher disease burden than MGUS and LC-MGUS but without MM or WM related organ damage^1^. Smoldering disease carries a higher risk of progression to active disease than MGUS. Retrospective data from the Mayo Clinic suggest that the risk of progression from SMM to MM is 10% per year for the first five years^9^, and that the risk of progression of SWM to WM is 60% within 10 years^10^.

Currently, consensus guidelines recommend indefinite follow-up in MGUS, SMM, and SWM. However, there is no data available from prospective studies or randomized trials regarding optimal clinical management^1,11–13^. Three recent observational studies from Sweden and the US have consistently demonstrated that individuals with known MGUS prior to the diagnosis of MM have 13–15% better overall survival in MM^14–16^. These observations indicate that clinical follow-up of precursor disease leads to earlier detection and diagnosis of MM, resulting in fewer patients presenting with symptomatic end-organ damage at the time of MM diagnosis, which may have contributed to the observed better overall survival.

In the clinical setting, the optimal timing of therapy in MM has been a subject of debate. Traditionally, therapy has been reserved for those with MM-related end-organ damage, however, in 2014 the definition of MM was expanded to also include myeloma-defining biomarkers in asymptomatic individuals^8^. With the advent of newer, more effective, and less toxic drugs, survival has improved dramatically in MM^17–19^. Three separate randomized controlled trials starting therapy at the stage of SMM have shown improved progression-free survival, and one study showed superior overall survival^20–22^. Importantly, these studies have shown more favorable toxicity profiles than earlier trials^23^. In light of these findings some authors now recommend early treatment in high-risk SMM^24,25^. However, only 2.7–6.0% of MM patients have previously identified precursor disease, which limits the implementation of early treatment in most MM patients^14,16^. This raises the question of whether population-based screening and follow-up of MGUS could improve the outcomes in MM by identifying candidates for early treatment. However, there is no evidence supporting the implementation of asymptomatic screening for MGUS, and screening is not currently recommended. To address this question, we have launched a population-based screening study with a subsequent randomized controlled trial (RCT) evaluating the risks and benefits of screening and follow-up of MGUS patients.

Here, we describe the design and recruitment of the Iceland Screens, Treats, or Prevents Multiple Myeloma study (iStopMM), a population-based screening study of MGUS and the disorders it precedes and RCT of follow-up strategies.

## Methods

### Approval

The study protocol, all information material, biobank, and questionnaires were approved by the Icelandic National Bioethics Committee (Number 16–022, date: 2016-04-26) with approval from the Icelandic Data Protection Agency. Access to national healthcare registries has been approved by the Icelandic Directorate of Health and the Icelandic Cancer Society. The study was preregistered on ClinicalTrials.gov (ClinicaTrials.gov identifier: NCT03327597).

### Recruitment and screening

The study’s inclusion criteria were being born in 1975 or earlier and residing in Iceland on the 9th of September 2016, as registered in the Icelandic National Registry. Eligible individuals were invited to participate in the iStopMM study (*n* = 148,711). A letter containing a detailed information brochure and consent form was mailed to them and an extensive campaign on social and conventional media was launched introducing the study to the Icelandic public. This campaign was followed by phone calls to those who had not yet signed up for the study. Participants could provide informed consent through three different mechanisms: (1) returning a signed informed consent form by mail, (2) registering electronically using a participation code included in the invitation letter, or (3) through a secure internet gateway provided by the Icelandic government (island.is), which is accessible to all residents through a secure electronic authentication process. The only exclusion criterion was previously known LP, other than MGUS.

**Fig. 1:**
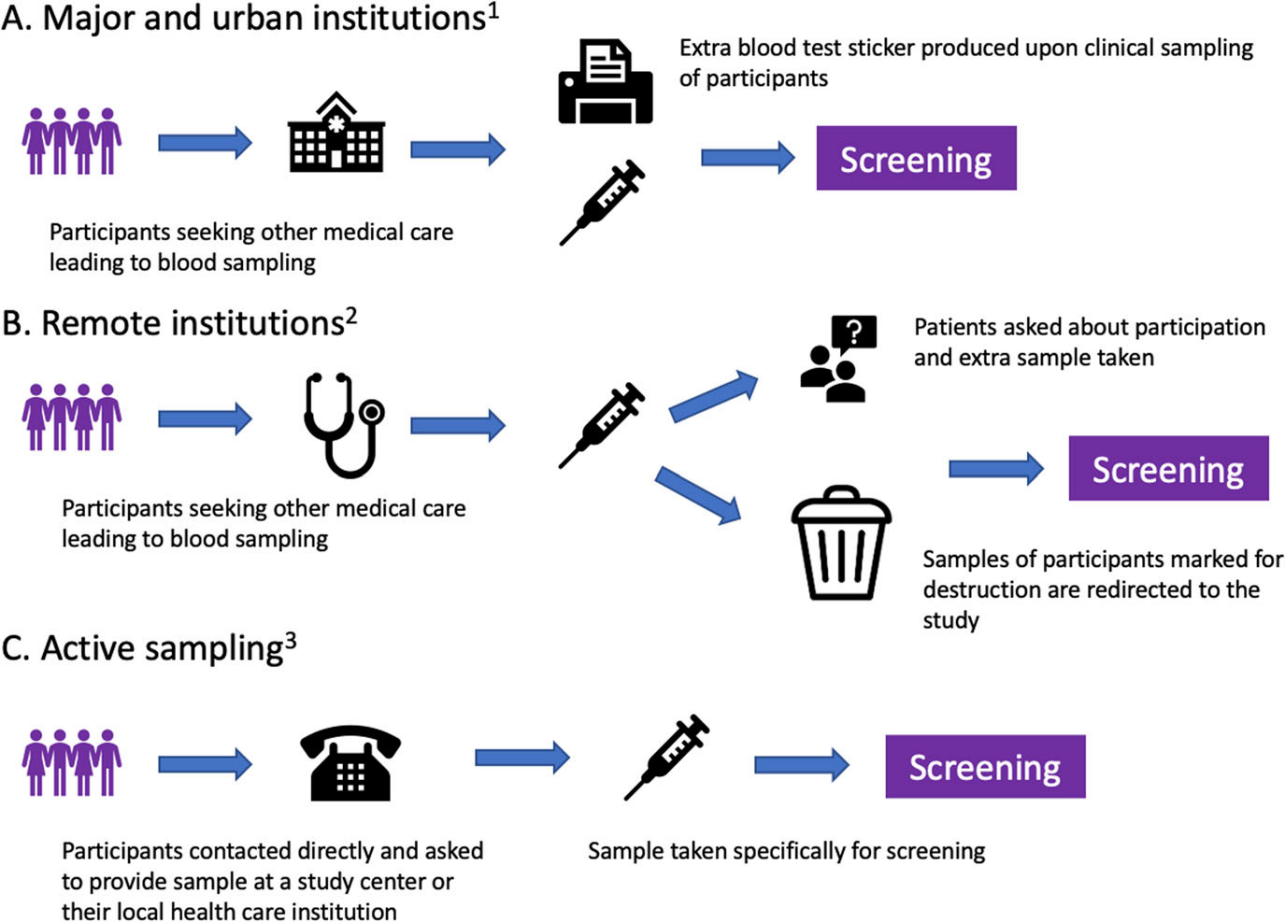
Methods of blood sample acquisition. **A** and **B** describe passive sampling starting during the fall of 2016, and **C** describes active sampling beginning during the fall of 2019. 1: Reykjavik Capital Area, Akureyri, Ísafjörður, Reykjanes Peninsula, Akranes, Healthcare Institution of Northern Iceland, Healthcare Institution of South Iceland, blood banks 2: Neskaupsstaður, Healthcare institution of West Iceland, Healthcare Institution of East Iceland. 3: Available for all Icelandic residents.

After enrollment, serum samples for screening are collected alongside the collection of blood during clinical care in the universal Icelandic healthcare system, including blood banks (Fig. 1). The study team in collaboration with Landspítali—The National University Hospital of Iceland (LUH), developed an electronic system linking participant data to the central laboratory network of all major and smaller urban healthcare institutions, which covers at least 92% of all Icelandic residents. The system notifies healthcare workers to take an extra blood sample for the study at the point of clinical blood sampling. For smaller rural institutions and private clinics, a manual system was developed whereby laboratory technicians crosslink left-over samples marked for destruction to registered participants and in some cases ask their patients if they are participants in the study and draw an additional sample for the study. To capture samples from participants who do not require clinical blood sampling, an active sampling drive was initiated after three years of passive sample collection.

All samples are sent to the clinical laboratory at LUH in Reykjavik, Iceland where serum is aliquoted into identical sample tubes and assigned an anonymous study identification number. The laboratory uses TC automation and aliquoter (Thermo Scientific®, MA, USA) for sample handling. Samples are then sent to The Binding Site laboratory in Birmingham, UK where all samples are screened for M protein by capillary zone electrophoresis (CZE; Helena Laboratories, Texas, USA) and for FLC, immunoglobulins (IgG, IgA, and IgM), and total protein by Freelite® and Hevylite® assays performed on an Optilite® turbidimeter (The Binding Site Group Ltd, Birmingham, UK). Immunofixation electrophoresis (IFE; Helena Laboratories, TX, USA) is performed on samples with clear or suspected M protein bands by CZE and/or abnormal FLC results. The CZE and IFE gels are assessed independently by at least two experienced observers.

### Randomization and study arms

**Fig. 2:**
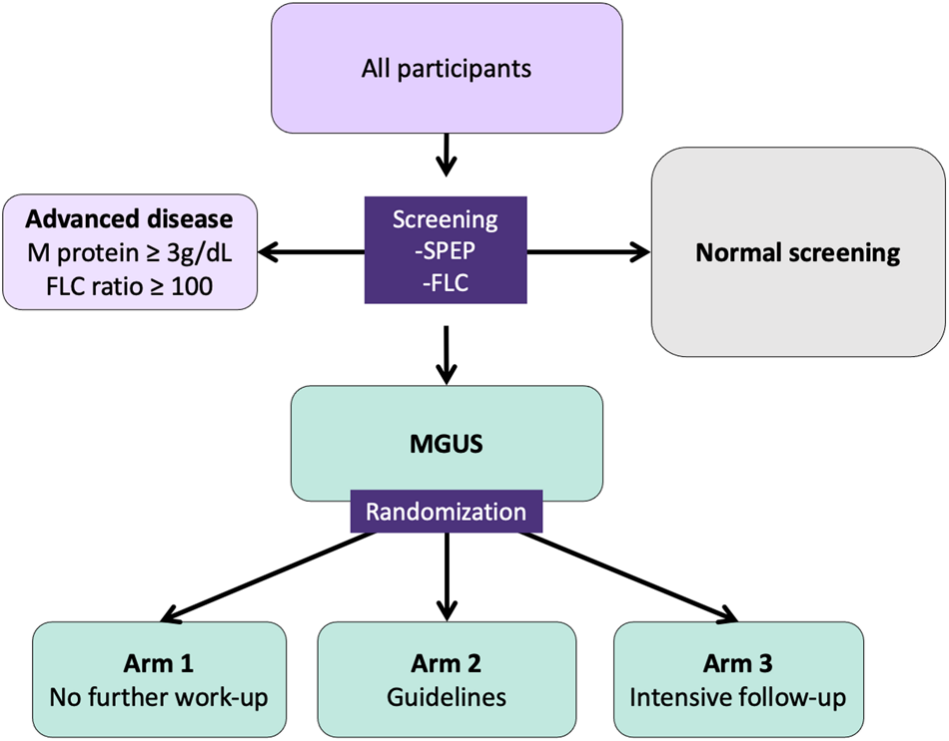
A flowchart outlining the study design for screening and randomization of individuals with MGUS. MGUS Monoclonal gammopathy of undetermined significance, SPEP Serum protein electrophoresis, FLC Free light chain assay, SMM Smoldering multiple myeloma, MM Multiple myeloma.

Participants with an M protein or pathological FLC results are considered eligible for the RCT and are randomized into three study arms in a dynamic, non-predetermined manner (Fig. 2). To avoid skewed distribution of high-risk MGUS and LC MGUS, randomization is carried out by blocks of having an M protein >1.5 g/dL and having LC-MGUS. Participants in arm 1 are not informed of their MGUS status and continue to receive conventional healthcare as if they had never been screened. Arm 2 follows current guidelines for follow-up, stratified by low and non-low risk MGUS^1^. Arm 3 follows a more intensive strategy that is not risk-stratified (see below).

Participants with an M protein ≥3.0 g/dL or an FLC ratio ≥100 are not eligible for randomization but are all called in for evaluation since they have, by definition, more advanced disease than MGUS^1,8,10^. Participants with previously diagnosed MGUS cannot be randomized to arm 1, as they are aware of their MGUS status, and are thus randomized to arms 2 or 3 and will not be included in comparisons with arm 1.

### Initial assessment and follow-up

Initial assessment and follow-up of participants in arms 2 and 3 and participants diagnosed with more advanced disease (SMM, SWM, MM, AL, or other LP) at screening is performed in the iStopMM study clinic in Reykjavík, Iceland. Temporary clinics are also regularly established in Akureyri, Ísafjörður, Húsavík, and Egilsstaðir for complete geographical coverage. All participants who are called into the clinic are seen by specialized study nurses and those with more advanced disease are also seen by a physician. The participants undergo a clinical interview and thorough clinical examination and are given detailed oral and written information about their diagnosis and prognosis.

Participants in arm 2 with non-IgM MGUS or LC-MGUS are stratified by having low-risk MGUS or not. These participants are then followed according to guidelines including plain skeletal surveys and bone marrow sampling for those with non-low risk MGUS or when clinically indicated^1^. All participants in arm 3 follow an intensive follow-up schedule regardless of risk, including bone marrow sampling and whole-body low-dose computerized tomography (WB-LDCT). Participants in arm 2 and 3 with IgM MGUS undergo a computerized tomography (CT) of the abdomen. Diagnostics and follow-up intervals for arms 2 and 3 are shown in Table 1. Participants with smoldering or active disease at baseline or later are followed according to guidelines. This includes intensive follow-up every 4 months or sooner if clinically indicated with annual bone marrow samples and WB-LDCT, as well as magnetic resonance imaging (MRI) if no bone lesions are seen on WB-LDCT. Participants who develop intermediate to high-risk SMM, MM, or other related disorders that require treatment are offered participation in a treatment trial (ClinicalTrials.gov identifier: NCT03815279) or referred to the hematology unit at LUH or Akureyri Hospital for evaluation, treatment, and follow-up.

**Table 1:** Clinical assessment, imaging, and laboratory studies included for participants in the different study arms of the iStopMM study as per protocol.

Test	Arm 2–low risk and LC-MGUS	Arm 2–non-low risk	Arm 3–All	SMM and SWM	MM and WM
Physical exam^a^	First visit	First visit	Each visit	Each visit	At diagnosis
*Blood sampling*					
SPEP FLC assay	Each visit	Each visit	Each visit	Each visit	At diagnosis
CBC	First visit	Each visit	Each visit	Each visit	At diagnosis
Total calcium Albumin Creatinine	First visit	First visit	Each visit	Each visit	At diagnosis
CRP LDH ß2M	–	–	Each visit	Each visit	At diagnosis
TnT pro-BNP	–	–	Annually	Annually	At diagnosis
*Bone marrow*					
Smear Biopsy	As clinically indicated	0 months Except if LC	0 and 60 months	Annually	At diagnosis
*Urine*					
Protein dipstick	First visit	First visit	–	–	–
UPEP	If positive dipstick or if previously abnormal	If positive dipstick or if previously abnormal	–	–	–
Albumin/creatinine ratio	–	–	Annually	Annually	At diagnosis
ECG	–	–	Annually	Annually	At diagnosis
*Imaging*					
WB-LDCT	–	–	0 and 60 months in LC- and non-IgM	Annually in LC- and non-IgM	At diagnosis of MM
Plain X-ray of bones	As clinically indicated	First visit in LC- and non-IgM	–	–	–
CT abdomen	–	First visit to IgM	0 and 60 months in IgM	Annually in IgM	At diagnosis of WM
MRI of bones	–	–	–	As clinically indicated	–
Follow-up	Every 2–3 years	Annual	Annual	Every 4–6 months	Single-visit

Note that additional sampling and imaging were permitted as clinically indicated and decided at regularly scheduled clinical decision meetings.

*SMM* smoldering multiple myeloma, *SWM* smoldering Waldenströms macroglobulinemia, *MM* multiple myeloma, *WM* Waldenströms macroglobulinemia, *SPEP* serum protein electrophoresis, *FLC* free light chains, *CBC* complete blood count, *CRP* C-reactive protein, *LDH* Lactate dehydrogenase, *ß2M* ß-2-microglobulin, *TnT* Troponin T, *pro-BPN* pro-Brain natriuretic peptide, *UPEP* Urine protein electrophoresis, *ECG* electrocardiogram, *WB-LDCT* whole-body low-dose computerized tomography, *CT* Computerized tomography, *MRI* magnetic resonance imaging, *LC* Light chain.

To detect AL, urine samples are tested for proteinuria in participants visiting the study clinic. In addition, participants in arm 3 and those with more advanced disease are tested for cardiac markers (Table 1). Those with significant proteinuria and decreased kidney function of unclear etiology are referred to a nephrologist for further evaluation. Those with abnormal cardiac markers not explained by known comorbidities are referred to a cardiologist for clinical evaluation and echocardiography. Bone marrow biopsies are stained with Congo red for the presence of amyloid fibrils in all these cases and another testing for AL is performed as clinically indicated.

After each visit, participant’s test results and clinical findings are thoroughly reviewed by the primary investigator and the clinic staff with respect to their disease status and progression at regular clinical decision meetings. Additional testing including repeat bone marrow sampling, imaging, blood sampling, or clinical evaluation is ordered as clinically indicated at or between protocol visits. Diagnoses of SMM, MM, SWM, WM, AL, and other LP are made according to current diagnostic criteria^1,8,26,27^.

### Imaging

Plain radiographs, WB-LDCT, and CT of the abdomen are performed in LUH and Akureyri Hospital. MRI is performed in LUH and Akureyri Hospital. All radiological images are reviewed independently by two physicians, one in specialty training and a senior radiologist at LUH. The radiological assessments are blinded and any discordance in findings is discussed and solved by the two physicians.

### Bone marrow samples

Bone marrow sampling is performed by study nurses that have been trained, both locally and in an accredited facility in the United Kingdom (The Royal Marsden Hospital, London, UK). Samples are collected as bone marrow smears and as trephine biopsies. Bone marrow smears are stained with Giemsa stain and jointly evaluated by two senior hematologists at LUH reporting the percentage of BMPCs or lymphoplasmacytic lymphocytes, lymphoid infiltrates, and sample quality. Trephine biopsies are stained with hematoxylin and eosin, as well as for CD138 before being evaluated by two senior hematopathologists at LUH. The sample with the higher percentage of BMPCs/lymphocytic infiltration at each sampling time is used to guide follow-up.

### Questionnaires

Immediately following informed consent, participants were asked to complete questionnaires on psychiatric symptoms (e.g., anxiety and depressive symptoms) and life satisfaction to establish a baseline prior to screening^28–30^. Throughout the study period, all participants, regardless of screening status, are asked to complete the same questionnaires electronically at predefined intervals, as well as additional questionnaires on psychiatric health, pain, neuropathic symptoms, and more (Table 2).

**Table 2:** Questionnaires sent to participants by email or answered at the study clinic.

Questionnaire	Subject	Validated?	All	Arm 1 and normal screening		Arm 2 and 3 and advanced disease^a^	
			At registration	One time	Annually	One time	Each visit
*Background*							
Anthropomorphic data	Weight, height etc.	NA		✓		✓	
Social history^b^	Socioeconomic status	NA		✓		✓	
Medical history^c^	Medical history			✓		✓	
Habits^d^	Environment	NA		✓		✓	
Industrial exposure	Environment	NA		✓		✓	
*Quality of life*							
PHQ9	Depression	Yes	✓		✓		✓
GAD-7	Anxiety	Yes	✓		✓		✓
SWLS	Quality of life	Yes	✓		✓		✓
Other questions of happiness and wellbeing	Quality of life	No	✓		✓		✓
SF-36	Health-related quality of life	Yes			✓		✓
PSS-10	Stress and anxiety	Yes			✓		✓
PCL-5 (MGUS specific)	PTSD from MGUS diagnosis	Yes					✓
PCL-5 (nonspecific)	PTSD other	Yes			✓		
*Symptoms*							
BPI	Pain	Yes			✓		✓
NSS	Neuropathy	Yes			✓		✓
DN4	Neuropathy	Yes			✓		✓
Symptoms of PMR	PMR	No			✓		✓
*Social background*							
MSPSS	Social support	Yes		✓		✓	
CD-RISC-10ICE	Resilience	Yes		✓		✓	
ACE	Childhood traumatic events	Yes		✓		✓	
LEC	Lifetime traumatic events	Yes		✓		✓	

Note that all participants were asked to answer four questionnaires when providing informed consent electronically or if they provided an email address in their written consent form.

Questionnaires were not sent to participants who did not provide an email address and were not called into the study.

*PHQ9* patient health questionnaire, *GAD-7* General anxiety disorder, *SWLS* satisfaction with life scale, *SF-36* 36-item short-form survey, *PSS-10* perceived stress scale, *PCL-5* post-traumatic stress disorder checklist for DSM-5, *BPI* brief pain inventory, *NSS* neuropathy symptom scale, *DN4* Douleur neuropathique. *PMR* polymyalgia rheumatica, *MSPSS* Multidimensional scale of social support, *CD-RISC-10ICE* Connor-Davidson resilience scale. *ACE* adverse childhood events. *LEC* Lifetime events checklist.

^✓^Showing the timing of the questionnaire in that row is the time/frequency assigned to that column.

^a^Including MM, WM, SMM, and SWM.

^b^Employment, marital status, education, income, and residence.

^c^Including obstetric history for women.

^d^Including smoking and alcohol intake.

Those who visit the study clinic (arms 2 and 3, and individuals with more advanced disease) answer more extensive questionnaires at each clinic visit and annually. Those who are randomized to arm 1 or are screened negative continue to receive the same annual questionnaires. One-time questionnaires, e.g., baseline characteristics, employment history, resilience, social support, and adverse childhood experiences are sent to all participants by email (Table 2).

Currently, 72 918 (90%) of all participants have provided their email addresses. All non-valid email addresses are reviewed by study staff and participants who visit the study clinic are asked to provide a valid email. Participants are reminded to answer the questionnaires in three separate emails.

### Registry crosslinking

Several national healthcare-related registries exist in Iceland that can be accurately crosslinked using a government-issued national identification number. Data from these registries are linked to all participants in the iStopMM study at least twice each year. The following registries are linked to the study datasets: (1) The Icelandic Cancer Registry includes information on all cancers diagnosed in Iceland. It has been mandatory for all physicians and pathologists to register diagnoses of cancer since 1955 and it is virtually complete with high diagnostic accuracy and timeliness^31^; (2) The Icelandic Causes of Death Registry includes all deaths in Iceland including the date and the presumed causes of death. Registration has been mandatory since 1971; (3) The Icelandic Prescription Medicines Registry includes all prescriptions, including whether the prescriptions were filled or not. in Iceland since 2002; (4) The Icelandic Hospital Discharge Registry includes all inpatient admissions in Iceland from 1999 with the dates of admission and discharge, as well as international classification of diseases (ICD) codes for the diagnoses made by treating physicians. The registry also includes outpatient visits at hospitals, including emergency rooms since 2010; (5) The Icelandic Registry of Primary Health Care Contacts includes all primary care visits and registered ICD-coded diagnoses for all primary care encounters in Iceland since 2004; (6) The Icelandic Central Laboratory Database comprises laboratory test results from all major clinical laboratories in Iceland stored in a central database since 1999, including all blood tests for participants prior to participation and during follow-up in the study; (7) All medical records at LUH, the only tertiary care medical center in Iceland and the general acute care hospital for the vast majority of Icelandic residents. This includes clinical notes, anthropometric data, written radiology and pathology reports, microbiology and virology test results, and all other documented clinical data.

### Biobanking

Blood samples drawn at each clinic visit are biobanked including cell-free plasma, serum, and plasma. Bone marrow samples are collected for biobanking in parallel to bone marrow sampling. Urine and blood in Blood-RNA tubes (PAXgene^TM^) tubes and in mononuclear cell preparation tubes (BD Vacutainer® CPT^TM^) are collected at sparser timepoints (Table 3). Samples are processed on-site and aliquoted at the study laboratory in Reykjavík, Iceland, and bone marrow samples separated into plasma and buffy coats. The bone marrow buffy coats from non-IgM MGUS and LC-MGUS are further separated into a plasma cell-enriched CD138+ fraction and a CD 138− fraction by Magnetic-activated cell sorting (MACS) using CD138 MicroBeads and an autoMACS pro cell separator (Miltenyi Biotec, Bergisch Gladbach, Germany). All cell fractions are cryopreserved and stored in liquid nitrogen. Other biobanking samples are frozen and stored in a secure state-of-the-art robotic biobanking facility in Reykjavík, Iceland, and cataloged using unique study identification numbers.

**Table 3:** Biosamples included in the study biobank and when they are obtained from participants.

Sample	Arm 2–Low risk	Arm 2–Non-low risk	Arm 3–All	SMM and SWM–4-month follow-up	SMM and SWM–6-month follow-up	MM and WM
*Bone marrow*						
Sorted and unsorted cells^a^	None	0 and 60 months	0 and 60 months	Annually	Annually	At diagnosis
Plasma	None	0 and 60 months	0 and 60 months	Annually	Annually	At diagnosis
*Blood*						
Cell-free plasma (EDTA tube)	0 months	Annually	Annually	Every 4 months	Every 6 months	At diagnosis
Plasma (Li-Hep tubes)	0 months	Annually	Annually	Every 4 months	Every 6 months	At diagnosis
Serum (SST tubes)	0 months	Annually	Annually	Every 4 months	Every 6 months	At diagnosis
Blood RNA (PaxGene® tube)	0 months	0 months	0 months	0 months	0 months	At diagnosis
Lymphocytes (CPT tube)	0 months	0 and 60 months	0 and 60 months	0 and 60 months	0 and 60 months	At diagnosis
*Urine*	0 months	0 months	0 months	Annually	Annually	At diagnosis

*SMM* smoldering multiple myeloma, *SWM* smoldering Walderströms macroglobulinemia, *MM* multiple myeloma, *WM* Waldenströms macroglobulinemia.

^a^Buffy coat from the bone marrow samples. Unsorted in IgM MGUS but stored as CD138+ and CD138− fractions using magnetic-activated cell sorting (MACS) in Non-IgM MGUS and LC-MGUS.

### Study monitoring

A study monitor was appointed to review the study protocol and regularly assessed the conduction of the study for compliance with relevant good clinical practice (GCP) principles. An independent data monitoring committee was established including two clinicians and a statistician that are not associated with the study. Interim analyses assessing safety and efficacy data are performed biannually. Additional interim analyses are scheduled when 500 subjects with MGUS have been followed for 6 months and when 100 participants with MGUS have died. When participants who have been randomized have been followed for five years, or if interim analysis shows a difference in the overall survival between arm 1 compared to arms 2 and 3, arm 1 will be discontinued. At that time the participants in arm 1 are unblinded to their MGUS status and offered a choice between randomization to arms 2 or 3, or clinical follow-up in the Icelandic healthcare system.

### Study endpoints

The primary endpoint of the study is the overall survival of individuals with MGUS receiving follow-up (arms 2 and 3) compared to those not receiving any follow-up within the study (arm 1) after 5 years of follow-up. Secondary endpoints are cause-specific survival due to MM or other LPs, psychiatric health and well-being, and cost-effectiveness of screening. In addition, study data will be crosslinked to registries and samples in the biobank providing a large dataset for future studies.

Assuming that 3360 individuals with MGUS are identified and the hazard ratio (HR) for the primary outcome is 0.81 as previously described^32^ the study has 77.2% power to reject the null hypothesis of HR = 1 at 5 years of follow-up and 89.3% power at 7 years of follow-up at an alpha level of 0.05.

## Results

A pilot recruitment phase was started in Akranes (population 7411) in Western Iceland on September 15th, 2016, to ensure that informational materials and processes of recruitment functioned as planned. After minor adjustments, the whole-nation recruitment phase commenced on November 15th, 2016, and continued until February 20th, 2018.

**Fig. 3:**
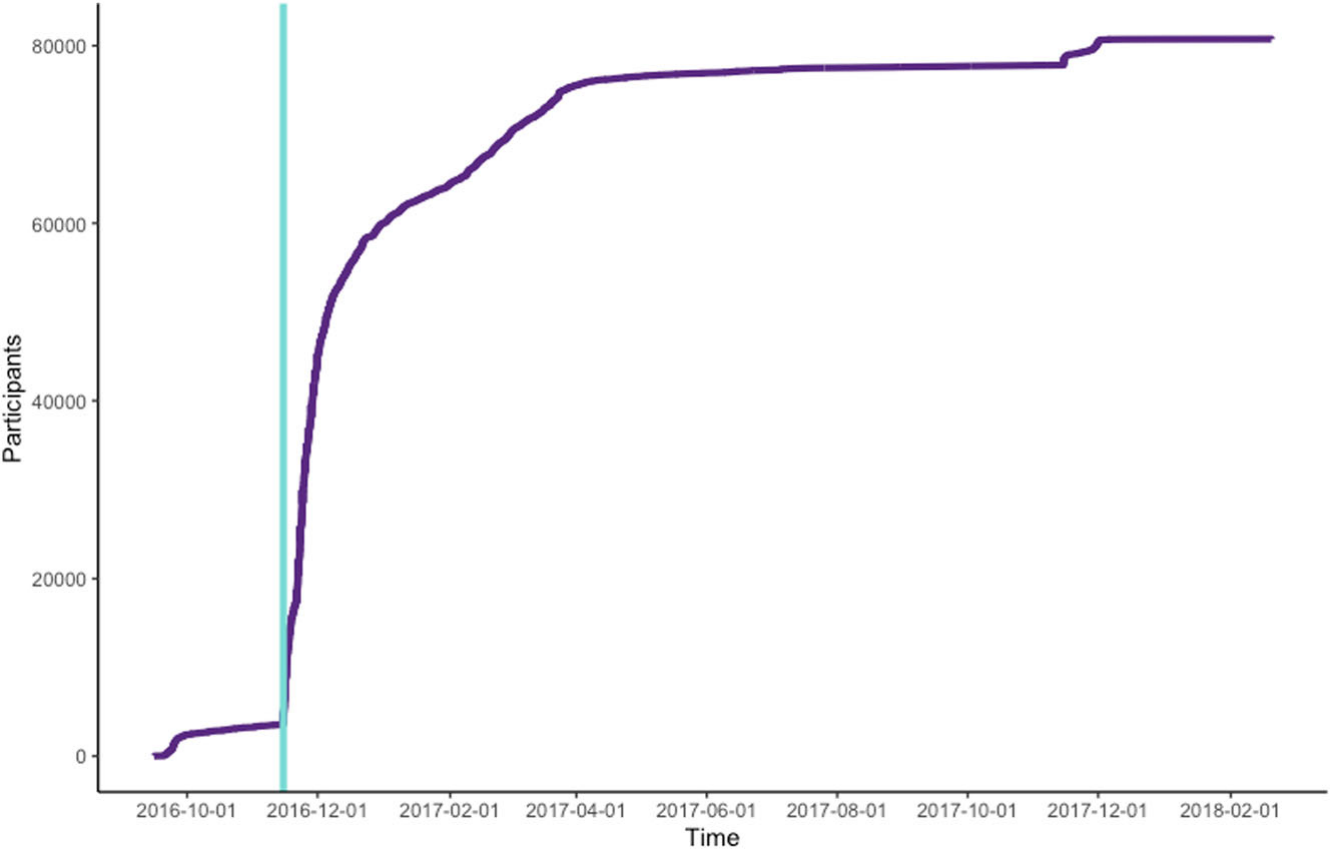
Participant enrollment over the recruitment period. The light green line represents the end of the pilot period and the initiation of nationwide recruitment.

A total of 148,704 individuals born in 1975 and earlier resided in Iceland when enrollment started, constituting the target population of the study. During the 15 months of recruitment, a total of 80,759 (54.3%) individuals provided informed consent for participation in the study (Fig. 3). Written informed consent was provided by 26% of participants while 74% provided informed consent electronically.

Of registered participants, 46% were male and 54% female constituting participation rates of 51% and 58%, respectively. Participation was highest (64%) among those between the ages of 60–79 but was lower (46%) in those between the ages of 40–49 and lowest (18%) among those over the age of 90 years old. The majority of participants (59%) were residents of the Reykjavik Capital Area with 18% and 23% of participants residing in other urban centers (more than 5000 inhabitants) and in rural areas, respectively. The participation rates were higher among those not residing in the Reykjavík Capital Area (60% versus 51% in the Reykjavík Capital Area; Table 4).

**Table 4:** The age, sex, and geographical distribution of participants and the target population, as well as available national registry data at the close of study recruitment.

	Registered participants	Target population
*n*	80,759	148,704
% females	54%	51%
median age^a^	59	57
Age range^a^	40–104	40–107
*Participation rate*		
All	54%	–
Males	51%	–
Females	58%	–
*Age group (male/female)*^a^		
40–49 (%)	21.2%/23.7%	27.4%/26.0%
50–59 (%)	27.7%/29.9%	29.4%/28.7%
60–69 (%)	28.4%/26.1%	23.4%/22.4%
70–79 (%)	16.6%/14.4%	12.9%/13.3%
80–89 (%)	5.7%/5.3%	6.0%/7.8%
>90 (%)	0.4%/0.5%	0.9%/1.8%
*Place of residence*		
Reykjavik Capital Area	58.7%	62.9%
Other urban centers^b^	17.5%	15.6%
Rural	23.3%	21.1%
Missing	0.6%	0.4%
Known MGUS^c^	246 (0.3%)	–
Previous LP^d^	548 (0.7%)	–
*Data from registries*^e^		
*n* hospital admissions	190,382	–
*n* primary care visits	8,187,805	–
*n* cancers diagnoses	10,328	–
*n* prescriptions	15,839,376	–

^a^Age at the time of study initiation on September 9th, 2016.

^b^Urban centers with >5000 inhabitants outside the Capital area.

^c^As registered before study enrollment in the Icelandic Cancer Registry since 1955, Icelandic Central Laboratory Database since 1999. and a registry of MGUS cases at Icelandic Private Clinics.

^d^As recorded before study enrollment in the Icelandic Cancer Registry since 1955.

^e^As recorded in national registries at the close of study enrollment on February 20th, 2018.

A total of 548 (0.7%) of participants had previously known LP before enrollment and were therefore excluded and 246 (0.3%) had previously known MGUS before enrollment. At the close of study enrollment on February 20th, 2018, a total of 190,382 hospital admissions since 1999, 8,187,805 primary health care visits since 2004, 10,328 cancer diagnoses since 1955, and 15,839,376 medication prescriptions in the national registries.

## Discussion

The iStopMM study is the first nationwide population-based, prospective screening study, and RCT among individuals with MGUS and the disorders it precedes. A total of 80,755 participants, 54.3% of the whole Icelandic population, born 1975 and earlier have enrolled in the iStopMM study. The high participation rate can be attributed to the extensive promotional effort undertaken in social and conventional media across Iceland where participation in scientific studies has historically been high^33–35^. In addition, using innovative solutions such as electronic informed consent and sampling parallel to clinical blood draws for screening, participants could easily sign-up and did not need to schedule a blood draw specifically for the study.

MGUS was first described as “benign gammopathy” by Dr. Jan Waldenström in 1960^36^ and later defined as MGUS by Dr. Robert Kyle in 1978^37^. Since then, screening studies in Olmstead county^2^ and the National Health and Nutrition Examination Survey in the US^38,39^, in Ghana^40^, and the PLCO-NCI Cancer Screening Trial^41^ have fundamentally changed our understanding of MGUS and the disorders it precedes. These studies have provided important evidence directing the course of clinical and basic science in the field and guided the management of individuals with MGUS. The iStopMM study builds upon these studies with nationwide screening and detailed clinical assessment and follow-up of individuals with MGUS within an RCT. Through this design, the iStopMM study aims to evaluate the potential harms and benefits of population-based screening while also providing evidence for the optimal diagnostic approach and follow-up of individuals with MGUS.

Guidelines currently recommend screening for cancers of the breast, cervix, colon, lungs, and prostate^42^. Cancer screening is controversial due to the high number of individuals needed to be screened to improve clinical outcomes and the high level of false-positive results that may lead to overtreatment, a lower sense of wellbeing, and even psychiatric illness^43^. In fact, a diagnosis of active cancer, including MM, has been associated with psychiatric disorders^44^ and suicide^45,46^. However, the role of screening in these outcomes is not known and such effects have not been shown to result from the diagnosis of pre-cancerous conditions like MGUS^47,48^. All participants of the iStopMM study are closely monitored for their psychiatric well-being using multiple psychometrically sound questionnaires. This will provide high-quality evidence on the potential psychological harms of MGUS screening that may have wider implications for cancer screening in general. Widely accepted criteria for when population-based disease screening is appropriate was developed by Wilson and Jungner in 1968^49^ and recently expanded further^50^. As detailed in Table 5, most of these criteria are already filled by MM. However, there are still important questions that need to be answered, most notably whether the benefits of screening outweigh the associated harms and costs. The results of the iStopMM study will provide answers to these outstanding questions on whether population-based screening is warranted in MM.

**Table 5:** Application of the Wilson and Jungner criteria and the additional recently proposed emerging criteria to multiple myeloma.

Criteria	Applies to MM?	Comment
*Original criteria*^49^		
The condition sought should be an important health problem	Yes	MM is the second most common hematological malignancy with 31,810 new cases and 12,770 attributed deaths in 2018 in the United States alone^53^
There should be an accepted treatment with recognized disease	Yes	Treatment for MM is widely available and international organizations recommending specific care for MM^54^
Facilities for diagnosis and treatment should be available	Yes	This at least applies to developed countries
There should be a recognizable or early symptomatic stage	Yes	MGUS and SMM are clearly established entities^1^ and precede all cases of MM^5,6^
There should be a suitable test or examination	Yes	SPEP, IFE, and FLC assays are sensitive and specific tests for MM and its precursors and can easily be repeated to confirm the diagnosis^55^
The test should be acceptable to the population	Yes	Screening is done by a blood test which is widely acceptable
The natural history of the condition, including development from latent to declared disease, should be adequately understood	Yes	Although there is still much to learn about the underlying pathogenesis of MM, a wealth of literature on the subject exists^56^. Furthermore, the natural history of MM and its development from precursor disorders is adequately understood with studies including decades of follow-up available^57^
There should be an agreed policy on whom to treat as patients	Yes	Although this is currently a moving target, there are clear guidelines on whom to treat, i.e., those with end-organ damage or myeloma defining events. In light of recent evidence, however, treatment might become available at even earlier stages^20,21,58^. If and when such early treatment is appropriate, there are institutions in place that will include such treatment in their guidelines
The cost of case-finding (including diagnosis and treatment of patients diagnosed) should be economically balanced in relation to possible expenditures on medical care as a whole	Unknown	There are currently no screening studies available for MM and its precursor conditions and a cost-benefit analysis is not available. This will be addressed as part of the iStopMM study
Case finding should be a continuing process and not a “once and for all” project	Yes	Since blood sampling for screening can be carried out at any time MM screening can be a continuing process
*Emerging screening criteria*^50^		
The screening program should respond to a recognized need	Yes	Although survival in MM has dramatically improved in recent years^17–19^ the disease remains a major burden on affected individuals and healthcare systems^59^
The objectives of screening should be defined at the outset	Yes	The objectives of screening for MM are clear: providing earlier treatment for MM
There should be a defined target population	Unknown	Currently, a well-defined target population for screening does not exist. This is addressed with regards to age, sex, and various other measures in the iStopMM study. However, due to the dominant white ethnicity of the Icelandic population, race cannot be addressed in the iStopMM study. Another study, the PROMISE study, focuses on the impact of screening in individuals of African descent. (ClinicalTrials.gov Identifier: NCT03689595)
There should be scientific evidence of screening program effectiveness	Unknown	The objective of the iStopMM study is to provide this evidence
The program should integrate education, testing, clinical services, and program management	Yes	There are excellent patent resources available in MM and its precursor disorders. Any screening program would be able to fulfill this criterion
There should be quality assurance, with mechanisms to minimize potential risks of screening	Yes	This organizational issue can be solved in MM screening since there are clear response criteria^60^ and accepted relevant endpoints like survival available for MM
The program should ensure informed choice, confidentiality, and respect for autonomy	Yes	This is a practical issue that does not require scientific proof of concept, although such proof is provided in the iStopMM trial
The program should promote equity and access to screening for the entire population	Yes	Since the cost of MM screening is relatively low and requires no specialized equipment at the point of patient care, equity in testing is therefore feasible. Follow-up for precursor disorders and treatment for MM can however be expensive and could lead to inequity in non-universal healthcare systems
Program evaluation should be planned from the outset	Yes	The practical issue of evaluation is possible for MM as proven by the methodology described above
The overall benefits of screening should outweigh the harm	Unknown	This is the principal study objective of the iStopMM study

Current clinical consensus guidelines for MGUS are not based on RCT data but rather on observational studies and expert opinions^1,11–13^. By conducting an RCT of different follow-up strategies, the iStopMM study aims to provide high-quality evidence for the optimal follow-up in MGUS. This includes the role of clinical assessment, questionnaires on symptoms, imaging, blood, bone marrow, and urine sampling. In addition, for research purposes, these clinical parameters are crosslinked to past and future testing in the Universal Icelandic healthcare, as well as health-related endpoints such as all cancers and death. Furthermore, novel testing modalities like next-generation flow cytometry of plasma cells in the blood and bone marrow^51^ and their microenvironment, mass spectrometry^52^, and single-cell, and germline genetics will be utilized to investigate their role in clinical management and to gain insight into the pathogenesis of MGUS and the biological processes involved in its progression to more advanced disorders. This is even further supplemented by the study´s extensive biobank, which includes blood, bone marrow, and urine samples collected repeatedly over the study period that can be retrieved at a later date for all participants or for participants of particular interest. With this extensive dataset and biobank, the iStopMM results will generate one of the most complete datasets on MGUS to date, providing unique opportunities for future studies.

The iStopMM study has some limitations. Firstly, the study is performed in Iceland which has a highly genetically homogenous white population and generalization of the study findings in non-white populations is somewhat limited. Secondly, by offering early treatment the natural history of MGUS progression to MM is affected. The main ethical issue of the study is that participants in arm 1 are not made aware of their MGUS status. These participants will not gain the potential benefits of screening but will also not be exposed to the potential harms of screening including psychological harms. These participants will continue receiving care in the universal Icelandic healthcare system and may be diagnosed there. Importantly, participants with markers of advanced disease at screening are not randomized to arm 1. Arm 1 will also be followed closely in regular interim analyses and will be unblinded if shown to have inferior survival.

In conclusion, using a novel and innovative recruitment methodology, including electronic informed consent and sampling parallel to clinical blood draws, as well as social and conventional media campaigns, over 80,000 individuals, more than half of the eligible Icelandic population, have enrolled in the iStopMM study. By population-based screening, follow-up of individuals with MGUS within an RCT, and early treatment in MM, the iStopMM study will generate large datasets and sample collections that will impact our basic understanding of MGUS and the disorders it precedes. Furthermore, it holds promise to fundamentally change the paradigm of MM treatment from late treatment in MM patients with end-organ damage to screening and early intervention, improving the overall survival and quality of life for patients worldwide.

## Supplementary information

checklist
