# Correction: Iceland screens, treats, or prevents multiple myeloma (iStopMM): a population-based screening study for monoclonal gammopathy of undetermined significance and randomized controlled trial of follow-up strategies

**DOI:** 10.1038/s41408-023-00814-w

**Published:** 2023-03-20

**Authors:** Sæmundur Rögnvaldsson, Thorvardur Jon Love, Sigrun Thorsteinsdottir, Elín Ruth Reed, Jón Þórir Óskarsson, Íris Pétursdóttir, Guðrún Ásta Sigurðardóttir, Brynjar Viðarsson, Páll Torfi Önundarson, Bjarni A. Agnarsson, Margrét Sigurðardóttir, Ingunn Þorsteinsdóttir, Ísleifur Ólafsson, Ásdís Rósa Þórðardóttir, Elías Eyþórsson, Ásbjörn Jónsson, Andri S. Björnsson, Gunnar Þór Gunnarsson, Runólfur Pálsson, Ólafur Skúli Indriðason, Gauti Kjartan Gíslason, Andri Ólafsson, Guðlaug Katrín Hákonardóttir, Manje Brinkhuis, Sara Lovísa Halldórsdóttir, Tinna Laufey Ásgeirsdóttir, Hlíf Steingrímsdóttir, Ragnar Danielsen, Inga Dröfn Wessman, Petros Kampanis, Malin Hultcrantz, Brian G. M. Durie, Stephen Harding, Ola Landgren, Sigurður Yngvi Kristinsson

**Affiliations:** 1 Faculty of Medicine, Univeristy of Iceland, Reykjavík, Iceland; 2 Dept of Hematology, Rigshospitalet, Copenhagen Denmark; 3 Landspítali University Hospital, Reykjavík, Iceland; 4 Faculty of Psychology, University of Iceland, Reykjavik, Iceland; 5 Akureyri Hospital, Akureyri, Iceland; 6 Faculty of Economics, University of Iceland, Reykjavik, Iceland; 7 The Binding Site, Birmingham, West Midlands UK; 8 Memorial Sloan Kettering Cancer Center, New York, NY USA; 9 Cedar-Sinai Samual Oschin Cancer Center, Los Angeles, CA USA; 10 Sylvester Comprehensive Cancer Center, University of Miami, Miami, FL USA

**Keywords:** Randomized controlled trials, Myeloma

Correction to: *Blood Cancer Journal* 10.1038/s41408-021-00480-w, published online 17 May 2021

In this article the author name Malin Hultcrantz was incorrectly written as Malin Hulcrantz.

The original article has been corrected.

